# Freehand Placement of a Transiliac‐Transsacral Screw for Fixation of Posterior Pelvic Ring Injuries

**DOI:** 10.1111/os.14326

**Published:** 2024-12-26

**Authors:** Guangping Liu, Zhiguang Chen, Wenhao Cao, Yubo Zheng, Jiaqi Li, Jie He, Changda Li, Hua Chen, Peifu Tang

**Affiliations:** ^1^ Department of Orthopaedics The Fourth Medical Center, Chinese PLA General Hospital Beijing China; ^2^ Department of Orthopaedics Zibo Central Hospital Zibo China

**Keywords:** entry point, fluoroscopy, pelvic fracture, posterior pelvic ring injury, Transiliac‐transsacral screw

## Abstract

**Objective:**

There are many advantages to stabilize the posterior pelvic ring injuries with a transiliac‐transsacral (TITS) screw percutaneously. To identify the correct entry point and insert a guidewire accurately for a TITS screw, we propose a method of specifying the optimal entry point, and introduce a technique of enabling freehand placement of a guidewire with fluoroscopic guidance.

**Methods:**

In this retrospective study, 116 patients who underwent pelvic CT scans and pelvic lateral radiographs at our institution from January 2020 to April 2022 were enrolled. The optimal entry point for a TITS screw was formulated in the strict mid‐sagittal CT plane, and then transferred to the pelvic lateral radiograph relying on the sacral cortexes which were easily visible even in the poor fluoroscopy. The relative position of this point to other anatomical markers was checked to confirm its feasibility as an entry point. With the method to locate the entry point, 18 patients suffered the posterior pelvic ring injuries were treated with TITS screws through hammering a reverse Kirschner wire (K‐wire) to insert a guidewire assisted by a canula, followed by the validation of the screw placement accuracy.

**Results:**

The transferred point in radiograph was consistently beneath the sacral alar slope, and located posteroinferior to the iliac cortical density (ICD) and anterosuperior to the sacral nerve root tunnel in all 116 patients. In clinical practice, 18 TITS screws were successfully placed in 18 patients without cortex violation. The average operative time for each screw was 20.11 ± 6.29 min, with an average of 14.11 ± 6.81 fluoroscopic shots per screw. At the 3‐month follow‐up, fracture healing was confirmed in all patients. The average Majeed score was 89.61 ± 6.90 at the final follow‐up.

**Conclusions:**

It's feasible to identify an entry point for a TITS screw based on the sacral cortexes, and hammering a reverse K‐wire assisted by a percutaneous kyphoplasty (PKP) canula is a safe and practical technique for guidewire insertion.

## Graphical abstract

This study introduced an approach for the freehand placement of a TITS screw with fluoroscopic guidance. The sacral cortexes, which are easily visible even in the poor fluoroscopy, served as the reference for identifying an entry point. For accurate guidewire insertion, a reverse K‐wire was hammered into the cancellous bone assisted with a PKP canula.

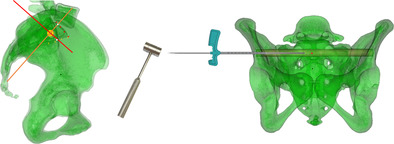

## 1 Introduction

Percutaneous sacroiliac (SI) screw fixation for posterior pelvic ring injuries is steadily gaining popularity [1, 2]. Compared to a conventional SI screw, a TITS screw can engage more cortexes, improve fixation stability and decrease screw loosening rate [1, 3, 4, 5]. It's practically more useful for bilateral injury, comminuted sacral fracture, fragility fracture, revision procedure, and spinopelvic dissociation [6, 7, 8, 9, 10]. Meanwhile, the outcomes and subjective pain scores do not be adversely affected for violating contralateral uninjured sacroiliac joint [11].

However, it is a technically demanding procedure to place a TITS screw with fluoroscopic guidance, because of the longer screw, adjacent neurovascular structures, and passing through bilateral sacral alar isthmus [12, 13]. Due to complex three‐dimensional pelvic anatomy, it's difficult to identify the correct entry point and the correct angle of screw trajectory. What is more complicated is the presence of sacral dysmorphism, which is seen in 35%–44% of adult patients [14, 15]. The rate of screw misposition has been reported to range from 2% to 38%, with an incidence of neurological injury up to 7.7% [16, 17, 18]. Not only the perforated screw but also the intraoperative guidewire was associated with iatrogenic nerve injuries.

Numerous techniques have been reported to improve the accuracy and safety of screw placement. Routt et al. emphasized the importance of fluoroscopic pelvic lateral view to locate the correct entry point [14]. Nevertheless, fluoroscopy often fails to clearly reveal the necessary anatomical landmarks due to bone overlap, obesity, poor bone quality and bowel gas [19]. To ensure correct entry and orientation of a guidewire, some customized templates were designed as sleeves [20, 21]. Although these were useful for guidewire insertion, the extra cost and preoperative time increased. A modified bent tip guidewire was usually employed to alter the initial guidewire misdirection [22]. However, since the guidewire was bent, it was potentially notched or bound by a cannulated drill.

In this study, we hypothesized the fluoroscopic pelvic lateral view could be mimicked by the strict mid‐sagittal CT image. On the basis of this hypothesis, (i) we formulated the optimal entry point in CT image, then verified the feasibility of transferring the entry point onto the pelvic lateral radiograph by referencing the sacral cortexes; (ii) following the entry point identification, we introduced a practical technique for freehand placement of a TITS screw.

## 2 Methods

### 2.1 Formulation and Verification of the Optimal Entry Point

#### 2.1.1 Patients

The study was approved by the institutional review committee (No. S2021‐340‐01). We retrospectively analyzed record of patients who underwent pelvic CT scans and pelvic lateral radiographs at our institution from January 2020 to April 2022. We formulated the optimal entry point of S1 segment as an illustration. Inclusive criteria: (1) the patient was ≥ 18 years old, (2) an intact pelvic posterior ring, (3) nondisplaced or minimally displaced fracture of the pelvic posterior ring, (4) the CT scan slice thickness ≤ 1 mm. Exclusive criteria: (1) the patient with deformed structure of pelvis caused by fracture or congenital diseases, (2) dysmorphic sacrum based on previously described criteria [14], (3) not strict lateral radiograph of the pelvis. According to the criteria, 116 patients were included in our study. There were 75 males and 41 females, with an average age of 46.1 years (ranging from 20 to 76 years, standard deviation 19.3 years). The group included 14 intact pelves, 16 pelvic anterior ring fractures and intact posterior ring, 29 nondisplaced or minimally displaced pelvic posterior ring fractures, 21 acetabular fractures, 15 femoral neck fractures, and 21 peritrochanteric fractures (Table 1).

**TABLE 1:** Patient demographics.

Characteristic	
Number	116
Age (mean ± SD) (year)	46.1 ± 19.3
Gender (Male/Female)	75/41
No of patients with intact pelves	14 (12%)
No of patients with only anterior ring fracture	16 (14%)
No of patients with non/minimally displaced posterior ring fracture	29 (25%)
No of patients with acetabular fracture	21 (18%)
No of patients with femoral neck fracture	15 (13%)
No of patients with peritrochanteric fracture	21 (18%)

#### 2.1.2 Formulating the Optimal Entry Point in CT Images

**FIGURE 1:**
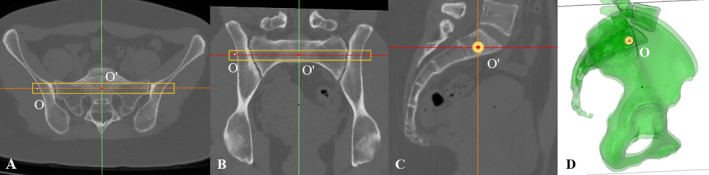
A 22‐year‐old female patient suffered pelvic anterior ring injury and intact posterior ring. The CT images were recut to obtain the strict axial plane (A), the strict coronal plane (B), and the strict mid‐sagittal plane (C). The cylinder (yellow rectangle) represented the most sufficient osseous corridor for a transiliac‐transsacral screw. Point O (white) was the intersection of the cylinder's axis and the outer iliac cortex, indicating the optimal entry point. Point O′ (red) was the intersection of the cylinder's axis and the strict mid‐sagittal plane. Point O and O′ were overlapped on the lateral view of the 3D pelvis (D).

We formulated the optimal entry point using an image editing software (Mimics version 21.0; Materialize, Belgium). CT data in Digital Imaging and Communications in Medicine (DICOM) format was imported into the Mimics software, and recut to obtain the strict axial plane (Figure 1A) and the strict coronal plane (Figure 1B). The sagittal planes were scrutinized to obtain the strict mid‐sagittal plane as shown in Figure 1C. A cylinder, simulating the osseous corridor for a TITS screw, was introduced in S1 segment. The cylinder was expanded and adjusted to engage the cortex of the nerve root tunnels posteriorly and sacral ala slope anteriorly. It was then checked to rule out cortex violation slice by slice through scrolling the sagittal planes. That means it possessed the maximum circular cross‐section without being extraosseous on any reconstructed planes. The cylinder represented the most sufficient osseous corridor for a TITS screw. The central axis of the cylinder indicated the optimal screw trajectory surrounding sufficient safe zone, and the intersection (O) of the axis and outer iliac cortex indicated the optimal entry point for a TITS screw. The intersection (O′) of the axis and the strict mid‐sagittal plane was the projection of point O on this plane. The two points (O and O′) were overlapped on the lateral view of the 3D pelvis (Figure 1D).

#### 2.1.3 Transferring the Entry Point Onto Radiograph and Verifying

**FIGURE 2:**
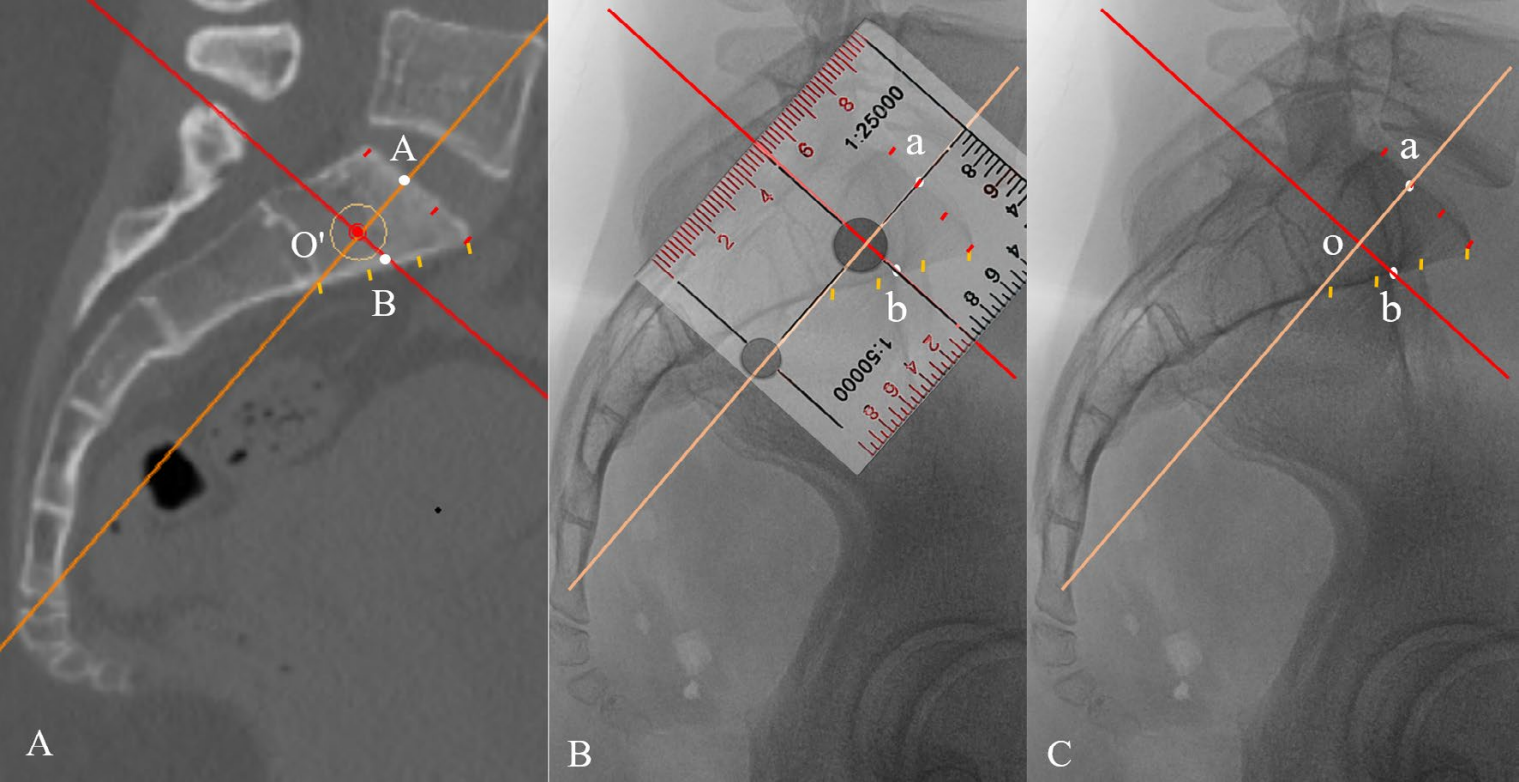
The optimal entry point was formulated in the strict mid‐sagittal plane using Mimics software (A) The red line (axial plane) was parallel to the upper endplate of S1 and intersected the anterior cortex of S1 body (point B). The orange line (coronal plane) was perpendicular to the red line and intersected the superior cortex of S1 body (point A). Point a and b were marked on the pelvic lateral radiograph corresponding to the position A and B. Two lines passing through point a and b were introduced with a coordinate ruler (B). The vertical crossing point o was verified as the optimal entry point for a transiliac‐transsacral screw (C).

Applying Mimics software, the strict mid‐sagittal plane was rotated around point O′ until the red line (axial plane) was parallel to the upper endplate of S1. The synchronized orange line (coronal plane) was consistently perpendicular to the red line. The two lines intersected the anterior and superior cortexes of S1 body respectively, designating point A and B. The anterior and superior cortexes were divided into three equal segments respectively, so as to identify the unique position A and B easily. Position O′ corresponded to position A and B (Figure 2A). On the lateral radiograph of the same pelvis, the anterior and superior cortexes of S1 body were similarly divided into three equal segments respectively. Then, we scrutinized and marked point a and b corresponding to the position A and B. Two lines passing through point a and b were introduced, and were perpendicular to each other with a coordinate ruler as shown in Figure 2B. Point o was the vertical crossing point (Figure 2C).

Whether the point o could serve as the entry point for a TITS screw intraoperatively needs to be verified. It's crucial to identify its relative position to landmarks on a plain radiograph, including the ICD, the sacral alar slope, and the sacral nerve root tunnel.

### 2.2 Clinical Practice

#### 2.2.1 Patients

From May 2022 to July 2023, 18 patients suffered pelvic posterior ring injuries were enrolled in the present study. Inclusion criteria were (1) the patient was ≥ 18 years old, (2) patients with a nondisplaced or minimally displaced fracture, (3) patients with a displaced fracture amenable to reduction with a closed maneuver. Exclusion criteria were (1) patients with an open or pathological pelvic fracture; (2) patients with a pelvic fracture or operation previously, (3) patients who can't tolerate anesthesia or operation, (4) patients associated with serious nerve or vascular injuries. The signed consent was obtained from each patient prior to surgery.

This study included 11 male and 7 female patients, with an average age of 43.7 ± 19.1 years. The fractures were classified according to the Tile classification [23], including five type B1, ten type B2, one type C1, and two type C2 fractures. The cause of injury included traffic accident in nine cases, fall from height in seven cases and crush injury in two cases (Table 2). All patients were fixed with a TITS screw by the same senior surgeon (G.L.).

**TABLE 2:** Demographic and clinical characteristics of 18 patients with pelvic posterior ring injury.

No	Age (years)	Gender	Type	Cause of injury	OP time (min)	Fluoroscopic shots	Guidewire attempts	Screw accuracy grade	Majeed score	Outcome
1	35	M	B2.2	FH	23	9	1	1	93	Excellent
2	41	M	B2.2	TA	16	8	1	1	95	Excellent
3	61	F	B1.2	CI	18	12	1	1	90	Excellent
4	42	F	B2.3	FH	26	16	1	1	85	Excellent
5	53	M	B1.2	FH	14	10	1	1	94	Excellent
6	45	F	C2.2	TA	27	32	1	2	86	Excellent
7	69	F	C1.3	TA	29	23	1	2	71	Good
8	49	M	B1.2	FH	14	11	1	1	92	Excellent
9	32	M	B2.2	CI	16	8	1	1	93	Excellent
10	79	F	B2.3	FH	17	13	1	1	85	Excellent
11	18	M	B2.3	TA	16	10	1	1	94	Excellent
12	79	F	B2.2	TA	21	12	1	1	92	Excellent
13	47	M	B2.3	TA	19	15	1	1	91	Excellent
14	45	M	C2.2	FH	35	28	1	1	75	Good
15	28	M	B1.2	FH	19	11	1	1	95	Excellent
16	21	M	B2.3	TA	26	14	1	1	93	Excellent
17	24	F	B2.2	TA	14	13	1	1	94	Excellent
18	19	M	B1.2	TA	12	9	1	1	95	Excellent

Abbreviations: CI, crush injury; FH, fall from height; OP, operation; TA, traffic accident.

#### 2.2.2 Surgical Procedure

The patient was placed on a radiolucent operating table under general anesthesia. The satisfactory fracture reduction was mandatory with the unlocking closed reduction technique (UCRT) [24], and confirmed with fluoroscopy. Then the C‐arm position and the degree of the beam were adjusted repeatedly and marked to obtain the optimal pelvic antero‐posterior view, inlet, outlet, and lateral pelvic views. This was done so the required views can be easily caught intraoperatively, thus minimizing the errant fluoroscopy frequency and unnecessary radiation exposure. Even so, it's still necessary to adjust the image intensifier throughout surgery to secure screw optimal positioning, once the situation changes during operation.

**FIGURE 3:**
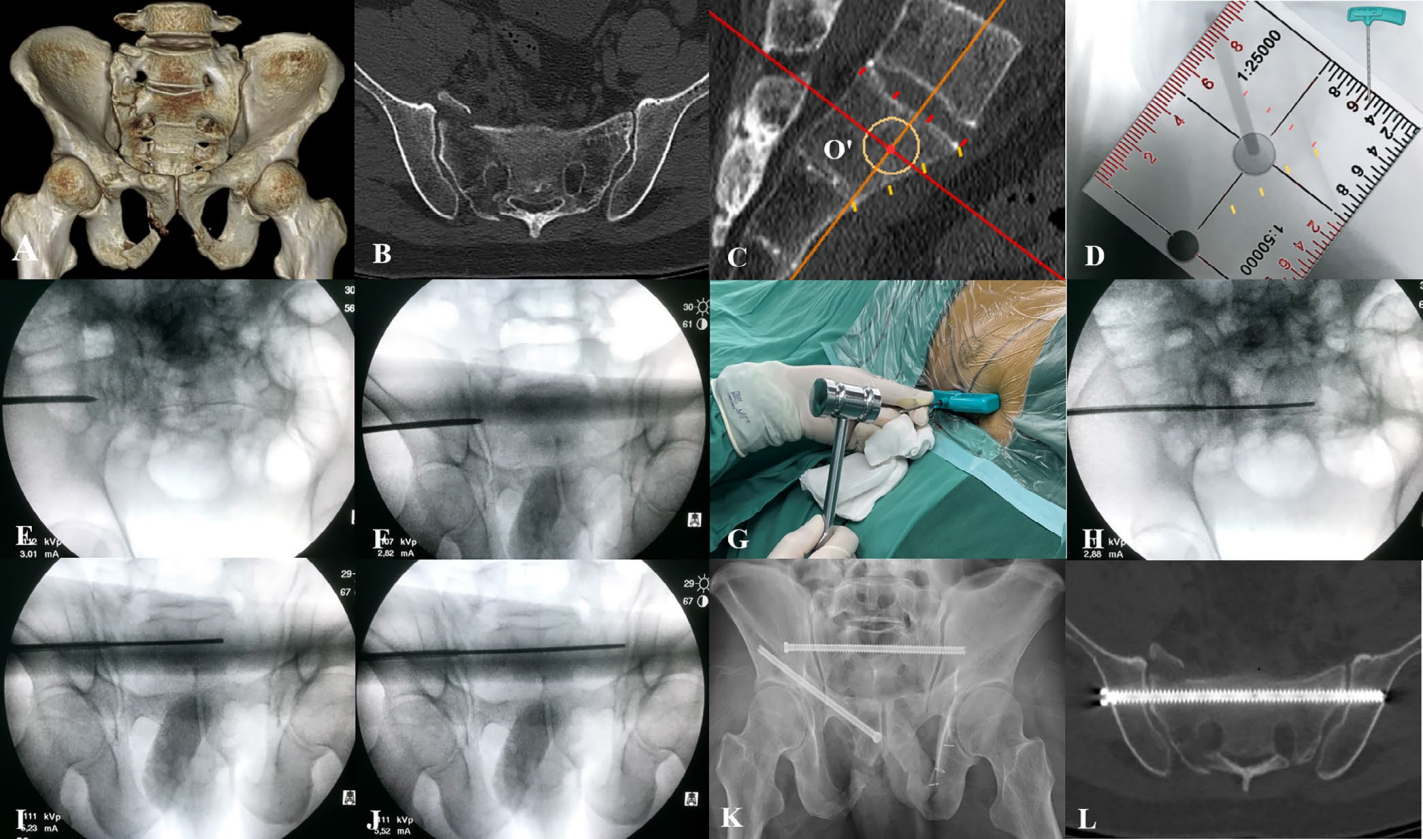
A 41‐year‐old man suffered a minimally displaced pelvic fracture (OTA‐61B2.2) (A, B) The optimal entry point was formulated in the strict mid‐sagittal plane (C), and transferred onto the lateral fluoroscopy (D). A percutaneous kyphoplasty canula was hammered to perforate the outer cortex of the ilium and pass through the ipsilateral sacroiliac joint (E, F). A reversed Kirschner wire was gently hammered and advanced (G–J). Postoperative pelvic radiograph and axial CT image showed the Grade‐1 placement of the transiliac‐transsacral screw (K, L).

After prepared and draped, the pelvic lateral view was obtained, and he anterior and superior cortexes of S1 body were divided into three equal segments on the screen, respectively. The reference points (a and b) were scrutinized and marked. Two lines, perpendicular to each other, were introduced based on the reference points with a coordinate ruler covered on the screen. The vertical crossing point was marked on the screen as the entry point. While maintaining the relative position of the image intensifier to the pelvis, a PKP canula was inserted against the bone surface, adjusted the tip of the canula until its image located at the entry point on the screen (Figure 3D). The canula was hammered to perforate the outer cortex of the ilium (Figure 3E). Then, the canula was adjusted for orientation (transverse within the bone and close to the anterior alar cortex on the inlet view, and cranial to the ventral foramen of the first sacral nerve root on the outlet view), and hammered to pass through the ipsilateral sacroiliac joint (Figure 3F). The included trocar was removed and the remaining canula served as a working tunnel for a guidewire. A reverse 2.5‐mm K wire was inserted into the tunnel, hammered gently and advanced within the cancellous bone, while being verified on the inlet and outlet views (Figure 3G–I). The wire advanced until hand‐feeling resistance was met, indicating the contralateral sacroiliac joint was reached (Figure 3J). A 5.0‐mm cannulated drill was then advanced and drilled over the wire. Accurate depth was measured with another K‐wire of equal length. A 6.5‐mm TITS screw was placed over the wire and confirmed the depth and length on bilateral oblique views tangential to the lateral iliac cortices. The incision was irrigated and sutured.

#### 2.2.3 Evaluation

The operation time, number of fluoroscopic shots and number of guidewire attempts related to the TITS screw placement were recorded. Postoperative radiographs and CT scans were performed to evaluate screw accuracy and grade as described by Cai et al. [25]. Follow‐up was conducted at 6 weeks, 3 months, 6 months and 1 year after surgery with regard to the complications, fracture‐healing, and function. The final clinical functional outcomes were evaluated by the Majeed score [26], which categorized as excellent (≥ 85 points), good (from 70 to 84 points), fair (from 55 to 69 points), or poor (< 55 points).

### 2.3 Statistical Analysis

The variables of patient data were summarized using standard descriptive statistics, such as the mean, standard deviation, or frequency. Statistical analysis was performed with the SPSS (version 26.0, IBM Corporation, USA).

## 3 Results

### 3.1 Formulation and Verification of the Optimal Entry Point

Upon verification, each individual point o was consistently beneath the sacral alar slope, and located posteroinferior relative to the ICD and anterosuperior relative to the sacral nerve root tunnel (Figure 2C). Therefore, it was proved feasible for simulating the entry point for TITS screw placement [14, 27]. We named this method “vertical crossing method”.

### 3.2 Clinical Practice

**FIGURE 4:**
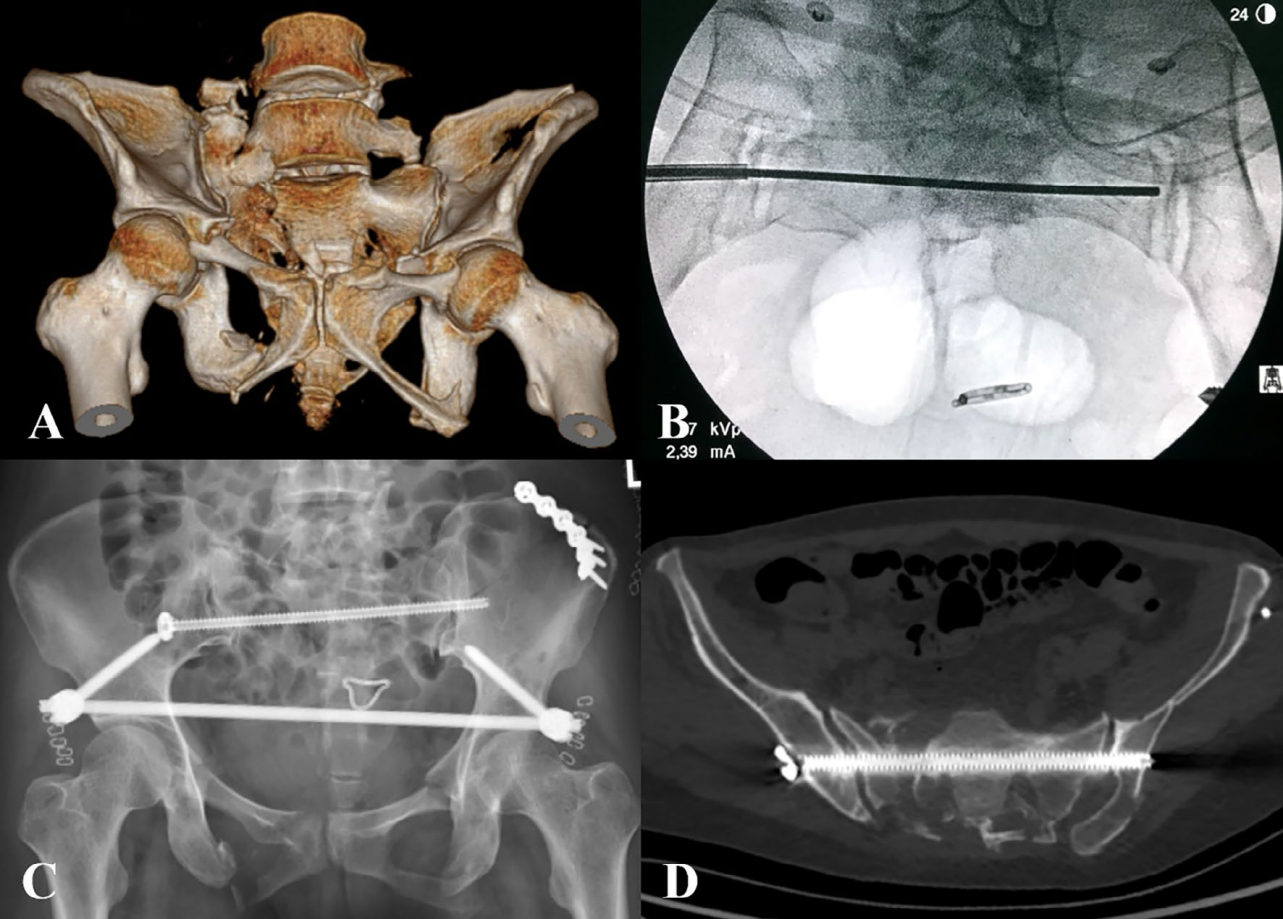
A 45‐year‐old woman suffered a displaced pelvic fracture (OTA‐61C2.2) (A). A reversed Kirschner wire was placed along the percutaneous kyphoplasty canula on the inlet view (B). Postoperative pelvic anteroposterior radiograph (C) and axial CT image showed the screw engaged the cortex of sacral nerve root tunnel (Grade‐2) (D).

A total of 18 TITS screws were successfully placed in 18 patients. The average operative time for each screw was 20.11 ± 6.29 min, with an average of 14.11 ± 6.81 fluoroscopic shots per screw. The first attempt to place the guidewire was successful for all patients. The screw accuracy and grade were evaluated in axial, coronal and sagittal plans of postoperative CT scans. All screws were safely located within the cancellous corridor. Sixteen screws (88.89%) were classified as Grade 1 and 2 screws (11.11%) were classified as Grade 2. The Grade 2 screws engaged the cortex of sacral nerve root tunnel but did not violate its cortical integrity (Figure 4). No patients had iatrogenic vascular or neurologic injuries after surgery. All patients were followed up, and the average follow‐up time was 13.59 ± 2.04 months. Fracture healing was confirmed in all patients at the 3‐month follow‐up. At the final follow‐up, the average Majeed score was 89.61 ± 6.90, with 16 patients having excellent function and two patients having good function. The proportion of excellent and good function was 100% (Table 2 was shown in the Appendix).

## 4 Discussion

Percutaneous TITS screw fixation after reduction has gained general acceptance to stabilize pelvic posterior ring injuries. However, freehand placement of a TITS screw remains a technical challenge for many orthopedic surgeons, particularly in identifying the entry point and guidewire insertion. Our study demonstrated that the vertical crossing method for identifying the entry point based on the sacral cortexes is reliable, and hammering a reverse K‐wire for precise guidewire insertion is secure. In our study, the percentage of correct screw position was greater than navigation cohort [28]. The average operative time per each screw placement was less than the time described previously, no matter in freehand or robot‐assisted manner (includes the time for preparing robot supporting facilities) [21, 29, 30]. Compared to previous robot‐assisted studies, there was no difference in the number of guidewire attempts and the rate of correct screw position, while our technique did not show an advantage in fluoroscopy shots and radiation exposure [31, 32].

### 4.1 Formulation of the Optimal Entry Point

For freehand placement, it is appreciated to plan with preoperative CT images in detail. Eastman et al. estimated the angles of inlet and outlet fluoroscopic views using the mid‐sagittal reconstruction image [33], while we mimicked the lateral view of the pelvis with the strict mid‐sagittal CT image. Upon performing the preoperative CT scan, the patient did not lie absolutely flat, or the body was distorted due to pain. The posture of the supine pelvis could impact the CT images and introduce measurement discrepancies. We recut and reconstructed images in any planner orientation to obtain strict sagittal planes, strict axial planes and strict coronal planes. These planes facilitate to formulate the most sufficient osseous corridor (the largest cylinder) for a screw, with the axis of the cylinder simulating the most optimal screw trajectory. Many studiers have also simulated secure screw placement in Mimics, but the influence of pelvic posture was not taken into account [34].

The present study introduced a point (O) situated on the axis of the cylinder. A screw through this point can tolerate a greater degree of deviation, because of the more sufficient safe zone surrounding the axis. The screw positioned at the edge of the cylinder easily violates the cortex for the screw diameter and deviation. In other word, a screw through this point allows a larger swing angle of the tip without cortical perforation. Therefore, this point in CT image can be deemed as the most optimal entry point for the TITS screw.

In surgical procedures with fluoroscopic guidance, previous studies trended to define a constant area for the entry point, rather than a specific point [35, 36]. Given the alar slope variation, each patient has a unique sacral alar anatomy [14]. That indicates that the most optimal entry point is individual for each patient. To transfer the optimal patient‐specific entry point to the lateral view, we selected sacral cortexes as references and landmarks, which could be easily visible even in the poor fluoroscopy. It was proved to be practical in clinical practice, and the vertical crossing method was validated as a reproducible method in our study.

### 4.2 Placement of a Guidewire for TITS Screw

After identifying the entry point, a guidewire should be placed in the appropriate direction and position. Chen et al. [20] designed an external template as a guidewire sleeve. It is useful for guidewire insertion, but the tip of the guidewire could easily slip on the oblique bone surface. A personalized locking template adhered to the bone would avoid this defect [21]. However, the additional time to design and print the template could prolong the waiting time for the definitive surgery. We apply a PKP cannula, which has been used to insert guidewire for LC2 screws [37], to better control the guidewire for TITS screw. It allows for a more stable start site, adjusting direction easily, and soft tissue protection.

A reverse K‐wire is gradually advanced as a guidewire with gentle hammer taps, and squeezes forward within the cancellous bone. Hammering a blunt end wire has been adopted to place pelvic brim screw [38] and LC2 screw [20]. We placed the guidewire for TITS screw in the similar manner. The blunt end minimizes the risk of cortical perforation [39], the rate of damage to vessels and nerves once inter‐fragment penetration. Additionally, that could also avoid nerve damage secondary to thermal injury and soft tissue being caught up in a rapid drilling manner [40].

The inner diameter of the PKP canula is 3.8 mm, larger than the diameter of the wire. That could facilitate the guidewire self‐adjustment when advancing. Furthermore, we selected a 2.5 mm wire, smaller than the 2.8 mm wire routinely employed in the literature [36, 41]. It is rigid enough to advance within the cancellous bone by gentle tapping, meanwhile it is more pliable than the 2.8 mm wire so as to spring off the cortex once engaging cortex.

**FIGURE 5:**
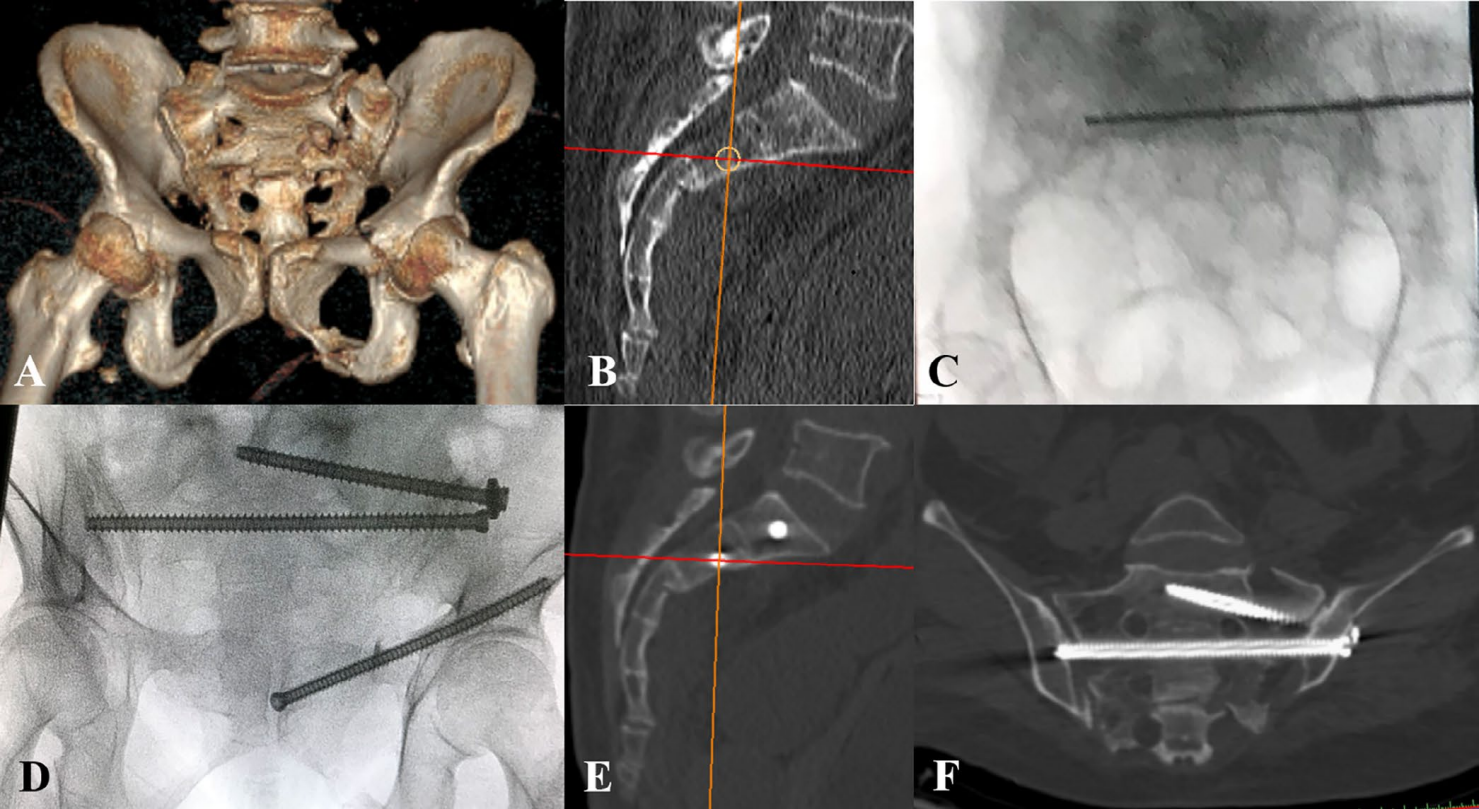
A 69‐year‐old woman suffered a minimally displaced pelvic fracture (OTA‐61C1.3) (A). The optimal entry point and the safe osseous corridor (orange circle) for transiliac‐transsacral screw of S2 were formulated in the strict mid‐sagittal plane (B). A reversed Kirschner wire was placed along the percutaneous kyphoplasty canula (C). Postoperative pelvic anteroposterior radiograph, strict mid‐sagittal CT image, and sacral coronal CT image showed the Grade‐2 placement of the screw (D–F).

Not every pelvis has sufficient safe zone in S1, while S2 more consistently offers safe zone for a TITS screw [42, 43]. Many studies favored S2 for screw placement in a dysmorphic sacrum [3, 19]. To place a TITS screw in S2, it is more difficulty because of less bone stock and more obscure fluoroscopy [44, 45]. In our clinical practice, it is also feasible to extrapolate our method and technique for S2 as shown in Figure 5. The yellow circle represents the sufficient osseous corridor for a TITS screw in S2 (Figure 5B), and the optimal entry point is the vertical crossing point of the orange line and the red line. When the orange line is rotated to intersect the posterosuperior corner of S2 body, the synchronized red line precisely passes through the anterosuperior and posteroinferior corners. Subsequently, the entry point is transferred and a screw is inserted. It is another pattern to identify the entry point based on the sacral cortexes.

### 4.3 Strengths and Limitations

To date, no other method has been reported for identifying the optimal patient‐specific entry point based on the sacral cortexes. The vertical crossing method we proposed has been validated in our study, even in the poor fluoroscopy. And hammering, rather than drilling, is a practical technique for secure guidewire insertion.

The study may have several limitations. One is the small sample size. The sample size will be expanded in a later study to further practice our method and procedure. Another limitation has no control group. Well‐designed prospective studies should be conducted to compare our method and technique with robotics or navigation assisted screw placement. Only one screw per sacral segment is also a limitation. Placing an additional screw in the same segment would complicate the surgery procedure.

### 4.4 Prospects of Clinical Application

In recent decades, although robotics and many kinds of navigation systems were applied to reduce malposition rate and radiation exposure [29, 46], freehand screw placement remains an elementary and essential skill for surgeons. It is an alternative once the artificial intelligence loses control intraoperatively. Our proposed method is concise and easy to grasp, and the technique requires no extensive equipment, thus facilitating widespread popularization. What's required is the preoperative planning for formulating the optimal patient‐specific entry point in CT scans.

### 4.5 Conclusions

The vertical crossing method based on the sacral cortexes is a reliable and reproducible method for identifying the optimal patient‐specific entry point, whatever for placing a TITS screw in S1 or S2. After determining the entry point, hammering a reverse K‐wire assisted with a PKP canula can facilitate secure guidewire insertion and improve the accuracy of screw placement.

## Author Contributions

All authors had full access to the data in the study and take responsibility for the integrity of the data and the accuracy of the data analysis. **Guangping Liu:** writing – original draft, methodology, investigation. **Zhiguang Chen:** formal analysis, writing – original draft. **Wenhao Cao:** investigation. **Yubo Zheng:** investigation. **Jiaqi Li:** investigation. **Jie He:** investigation. **Changda Li:** formal analysis. **Hua Chen:** conceptualization, methodology, writing – review and editing. **Peifu Tang:** supervision.

## Ethics Statement

All procedures performed in the study were in accordance with the ethical standards of the institutional. Informed consent was obtained from all individual participants.

## Conflicts of Interest

The authors declare no conflicts of interest.

## References

[1] R. Cintean , C. Fritzsche , I. Zderic , B. Gueorguiev‐Rüegg , F. Gebhard , and K. Schütze , “Sacroiliac Versus Transiliac–Transsacral Screw Osteosynthesis in Osteoporotic Pelvic Fractures: A Biomechanical Comparison,” European Journal of Trauma and Emergency Surgery 49, no. 6 (2023): 2553–2560. 37535095 10.1007/s00068-023-02341-6 PMC10728224

[2] E. T. Araiza , S. Medda , J. F. Plate , et al., “Comparing the Efficiency, Radiation Exposure, and Accuracy Using C‐Arm Versus O‐Arm With 3D Navigation in Placement of Transiliac–Transsacral and Iliosacral Screws: A Cadaveric Study Evaluating an Early Career Surgeon,” Journal of Orthopaedic Trauma 34, no. 306 (2020): 302–306. 32433194 10.1097/BOT.0000000000001724

[3] J. M. Conflitti , M. L. Graves , and M. L. Chip Routt, Jr. , “Radiographic Quantification and Analysis of Dysmorphic Upper Sacral Osseous Anatomy and Associated Iliosacral Screw Insertions,” Journal of Orthopaedic Trauma 24, no. 10 (2010): 630–636, 10.1097/BOT.0b013e3181dc50cd. 20871251

[4] J. G. Eastman , T. J. Shelton , M. L. C. Routt , and M. R. Adams , “Posterior Pelvic Ring Bone Density With Implications for Percutaneous Screw Fixation,” European Journal of Orthopaedic Surgery and Traumatology 31, no. 2 (2020): 383–389. 32902718 10.1007/s00590-020-02782-4

[5] W. Zhou , J. Chen , X. Pei , et al., “Incidence of and Risk Factors for Screw Loosening After Iliosacral Screw Fixation for Posterior Pelvic Ring Injury,” Orthopaedic Surgery 15, no. 7 (2023): 1814–1822. 37345455 10.1111/os.13763 PMC10350383

[6] J. G. Eastman , R. J. Kuehn , and M. L. Chip Routt, Jr. , “Useful Intraoperative Technique for Percutaneous Stabilization of Bilateral Posterior Pelvic Ring Injuries,” Journal of Orthopaedic Trauma 32, no. 5 (2018): e191–e197, 10.1097/BOT.0000000000001047. 29683436

[7] M. J. Gardner and M. L. Routt, Jr. , “Transiliac‐transsacral screws for posterior pelvic stabilization,” Journal of Orthopaedic Trauma 25, no. 6 (2011): 378–384, 10.1097/BOT.0b013e3181e47fad. 21577075

[8] P.‐H. Chen , C.‐Y. Chen , K.‐C. Lin , and C.‐J. Hsu , “Quantification of the Safe Zone of the First to Third Sacral Segments for Transiliac–Transsacral Screw Fixation in Normal and Dysmorphic Sacra,” Orthopedics 47, no. 1 (2024): e13–e18. 37276441 10.3928/01477447-20230531-06

[9] J. B. Walker , S. M. Mitchell , S. D. Karr , J. A. Lowe , and C. B. Jones , “Percutaneous Transiliac–Transsacral Screw Fixation of Sacral Fragility Fractures Improves Pain, Ambulation, and Rate of Disposition to Home,” Journal of Orthopaedic Trauma 32, no. 9 (2018): 452–456. 29916895 10.1097/BOT.0000000000001243

[10] E. Sevillano‐Perez , M. Prado‐Novoa , S. Postigo‐Pozo , A. Peña‐Trabalon , and E. Guerado , “L4 Fixation Is Not Necessary in L5‐Iliac Spinopelvic Fixation After Trauma, but Coadjutant Transilio‐Transsacral Fixation Is,” Injury 55, no. 3 (2024): 111378. 38309085 10.1016/j.injury.2024.111378

[11] S. W. Mardam‐Bey , M. J. Beebe , J. C. Black , et al., “The Effect of Transiliac‐Transsacral Screw Fixation for Pelvic Ring Injuries on the Uninjured Sacroiliac Joint,” Journal of Orthopaedic Trauma 30, no. 9 (2016): 463–468, 10.1097/BOT.0000000000000622. 27144820

[12] C. S. Day , M. J. Prayson , T. E. Shuler , J. Towers , and G. S. Gruen , “Transsacral Versus Modified Pelvic Landmarks for Percutaneous Iliosacral Screw Placement—a Computed Tomographic Analysis and Cadaveric Study,” American Journal of Orthopedics (Belle Mead, N.J.) 29, no. S9 (2000): 16–21. 11011775

[13] D. A. McLaren , G. A. Busel , H. R. Parikh , et al., “Corridor‐Diameter‐Dependent Angular Tolerance for Safe Transiliosacral Screw Placement: An Anatomic Study of 433 Pelves,” European Journal of Orthopaedic Surgery and Traumatology 31, no. 7 (2021): 1485–1492. 33649991 10.1007/s00590-021-02913-5

[14] M. L. Routt, Jr. , P. T. Simonian , S. G. Agnew , and F. A. Mann , “Radiographic Recognition of the Sacral Alar Slope for Optimal Placement of Iliosacral Screws: A Cadaveric and Clinical Study,” Journal of Orthopaedic Trauma 10, no. 3 (1996): 171–177. 8667109 10.1097/00005131-199604000-00005

[15] M. J. Gardner , S. Morshed , S. E. Nork , W. M. Ricci , and M. L. Chip Routt, Jr. , “Quantification of the Upper and Second Sacral Segment Safe Zones in Normal and Dysmorphic Sacra,” Journal of Orthopaedic Trauma 24, no. 10 (2010): 622–629. 20871250 10.1097/BOT.0b013e3181cf0404

[16] M. M. Hadeed , D. Woods , J. Koerner , K. E. Strage , C. Mauffrey , and J. A. Parry , “Risk Factors for Screw Breach and Iatrogenic Nerve Injury in Percutaneous Posterior Pelvic Ring Fixation,” Journal of Clinical Orthopaedics and Trauma 33 (2022): 101994. 36061971 10.1016/j.jcot.2022.101994 PMC9436800

[17] M. Pishnamaz , T. Dienstknecht , B. Hoppe , et al., “Assessment of Pelvic Injuries Treated With Ilio‐Sacral Screws: Injury Severity and Accuracy of Screw Positioning,” International Orthopaedics 40, no. 7 (2016): 1495–1501. 26260867 10.1007/s00264-015-2933-1

[18] J. Zwingmann , O. Hauschild , G. Bode , N. P. Südkamp , and H. Schmal , “Malposition and Revision Rates of Different Imaging Modalities for Percutaneous Iliosacral Screw Fixation Following Pelvic Fractures: A Systematic Review and Meta‐Analysis,” Archives of Orthopaedic and Trauma Surgery 133, no. 9 (2013): 1257–1265. 23748798 10.1007/s00402-013-1788-4

[19] T. Mendel , H. Noser , J. Kuervers , F. Goehre , G. O. Hofmann , and F. Radetzki , “The Influence of Sacral Morphology on the Existence of Secure S1 and S2 Transverse Bone Corridors for Iliosacroiliac Screw Fixation,” Injury 44, no. 12 (2013): 1773–1779. 24004615 10.1016/j.injury.2013.08.006

[20] K. Chen , S. Yao , F. Yang , et al., “Minimally Invasive Screw Fixation of Unstable Pelvic Fractures Using the “Blunt End” Kirschner Wire Technique Assisted by 3D Printed External Template,” BioMed Research International 2019 (2019): 1–9. 10.1155/2019/1524908 PMC6854157 31772932

[21] C. Wu , B. Zeng , J. Deng , et al., “Finite Element Analysis and Transiliac‐Transsacral Screw Fixation for Posterior Pelvic Ring With Sacrum Dysplasia,” Orthopaedic Surgery 15, no. 1 (2022): 337–346. 36424734 10.1111/os.13585 PMC9837241

[22] J. A. Scolaro and M. L. Routt , “Intraosseous Correction of Misdirected Cannulated Screws and Fracture Malalignment Using a Bent Tip 2.0 Mm Guidewire: Technique and Indications,” Archives of Orthopaedic and Trauma Surgery 133, no. 7 (2013): 883–887. 23589066 10.1007/s00402-013-1740-7

[23] M. Tile , “Pelvic Ring Fractures: Should They Be Fixed?,” Journal of Bone and Joint Surgery. British Volume (London) 70, no. 1 (1988): 1–12. 10.1302/0301-620X.70B1.3276697 3276697

[24] H. Chen , Q. Zhang , Y. Wu , et al., “Achieve Closed Reduction of Irreducible, Unilateral Vertically Displaced Pelvic Ring Disruption With an Unlocking Closed Reduction Technique,” Orthopaedic Surgery 13, no. 3 (2021): 942–948. 33817995 10.1111/os.12958 PMC8126934

[25] H. Cai , R. Zhang , Y. Yin , J. Li , Z. Hou , and Y. Zhang , “Specifying the Starting Point for S1 Iliosacral Screw Placement in the Dysmorphic Sacrum,” Journal of Bone and Joint Surgery 106, no. 2 (2024): 129–137. 10.2106/JBJS.23.00397 37992198

[26] S. A. Majeed , “Grading the Outcome of Pelvic Fractures,” Journal of Bone and Joint Surgery. British Volume (London) 71, no. 2 (1989): 304–330. 10.1302/0301-620X.71B2.2925751 2925751

[27] Z.‐H. Zheng , F. Xu , Z.‐Q. Luo , et al., “A Useful Intraoperative Technique for Transiliac‐Transsacral Screws: A Point‐To‐Point Coaxial Guide Apparatus,” Journal of Orthopaedic Surgery and Research 16, no. 1 (2021): 89. 33509244 10.1186/s13018-021-02239-2 PMC7845130

[28] J. Zwingmann , G. Konrad , E. Kotter , N. P. Südkamp , and M. Oberst , “Computer‐Navigated Iliosacral Screw Insertion Reduces Malposition Rate and Radiation Exposure,” Clinical Orthopaedics and Related Research 467, no. 7 (2009): 1833–1838. 19034594 10.1007/s11999-008-0632-6 PMC2690740

[29] C. Zhao , G. Zhu , Y. Wang , and X. Wu , “TiRobot‐Assisted Versus Conventional Fluoroscopy‐Assisted Percutaneous Sacroiliac Screw Fixation for Pelvic Ring Injuries: A Meta‐Analysis,” Journal of Orthopaedic Surgery and Research 17, no. 1 (2022): 525. 36471345 10.1186/s13018-022-03420-x PMC9721051

[30] P. Wang , K. Yang , H. Qi , et al., “Multimodal Neuroelectrophysiological Monitoring Combined With Robot‐Assisted Placement of a Transiliac–Transsacral Screw for the Treatment of Transforaminal Sacral Fractures,” BioMed Research International 2022 (2022): 1–9. 10.1155/2022/3383665 PMC9338859 35915799

[31] W. Han , T. Zhang , Y. G. Su , et al., “Percutaneous Robot‐Assisted Versus Freehand S2 Iliosacral Screw Fixation in Unstable Posterior Pelvic Ring Fracture,” Orthopaedic Surgery 14, no. 2 (2021): 221–228. 34904387 10.1111/os.13056 PMC8867425

[32] J. Q. Wang , Y. Wang , Y. Feng , et al., “Percutaneous Sacroiliac Screw Placement: A Prospective Randomized Comparison of Robot‐Assisted Navigation Procedures With a Conventional Technique,” Chinese Medical Journal 130, no. 21 (2017): 2527–2534. 29067950 10.4103/0366-6999.217080 PMC5678249

[33] J. G. Eastman and M. L. Routt, Jr. , “Correlating Preoperative Imaging With Intraoperative Fluoroscopy in Iliosacral Screw Placement,” Journal of Orthopaedics and Traumatology 16, no. 4 (2015): 309–316. 26195031 10.1007/s10195-015-0363-x PMC4633422

[34] K. R. Renate , V. E. Elisabeth , S. A. A. Maria , S. K. Sabine , R. W. Renate , and H. G. G. Maria , “Three‐Dimensional Morphometry of the First Two Sacral Segments and Its Impact on Safe Transiliac‐Transsacral Screw Placement,” Injury 52, no. 10 (2021): 2959–2967. 34275644 10.1016/j.injury.2021.06.029

[35] J. Tidwell , R. Cho , J. S. Reid , H. Boateng , C. Copeland , and E. Sirlin , “Percutaneous Sacroiliac Screw Technique,” Journal of Orthopaedic Trauma 30, no. 2 (2016): S19–S20. 27441927 10.1097/BOT.0000000000000606

[36] P. M. Rommens , E. M. Nolte , J. Hopf , D. Wagner , A. Hofmann , and M. Hessmann , “Safety and Efficacy of 2D‐Fluoroscopy‐Based Iliosacral Screw Osteosynthesis: Results of a Retrospective Monocentric Study,” European Journal of Trauma and Emergency Surgery 47, no. 6 (2020): 1687–1698. 32296862 10.1007/s00068-020-01362-9 PMC8629807

[37] J. Scherer , P. Guy , K. A. Lefaivre , H.‐C. Pape , C. M. L. Werner , and G. Osterhoff , “Guide Wire Insertion for Percutaneous LC2 Screws in Acetabular and Pelvic Ring Fixation Using a Transpedicular Working Cannula,” Injury 48, no. 10 (2017): 2360–2364. 28859845 10.1016/j.injury.2017.08.049

[38] N. C. Tejwani , D. Raskolnikov , T. McLaurin , and R. Takemoto , “The Role of Computed Tomography for Postoperative Evaluation of Percutaneous Sacroiliac Screw Fixation and Description of a Safe Zone,” American Journal of Orthopedics (Belle Mead, N.J.) 43, no. 11 (2014): 513–516. 25379748

[39] L. Zhang , P. Yin , W. Zhang , et al., “An Effective and Feasible Method, “Hammering Technique,” for Percutaneous Fixation of Anterior Column Acetabular Fracture,” BioMed Research International 2016 (2016): 1–6. 10.1155/2016/7151950 PMC4963568 27493962

[40] D. Wagner , L. Kamer , T. Sawaguchi , et al., “Space Available for Trans‐Sacral Implants to Treat Fractures of the Pelvis Assessed by Virtual Implant Positioning,” Archives of Orthopaedic and Trauma Surgery 139, no. 10 (2019): 1385–1391. 31111201 10.1007/s00402-019-03204-9

[41] C.‐S. Chon , J.‐H. Jeong , B. Kang , H. S. Kim , and G.‐H. Jung , “Computational Simulation Study on Ilio‐Sacral Screw Fixations for Pelvic Ring Injuries and Implications in Asian Sacrum,” European Journal of Orthopaedic Surgery and Traumatology 28, no. 3 (2017): 439–444. 29027586 10.1007/s00590-017-2061-2

[42] E. Gautier , R. Bächler , P. F. Heini , and L. P. Nolte , “Accuracy of Computer‐Guided Screw Fixation of the Sacroiliac Joint,” Clinical Orthopaedics and Related Research 393 (2001): 310–317. 10.1097/00003086-200112000-00036 11764364

[43] A. F. Hinsche , P. V. Giannoudis , and R. M. Smith , “Fluoroscopy‐Based Multiplanar Image Guidance for Insertion of Sacroiliac Screws,” Clinical Orthopaedics and Related Research 395 (2002): 135–144. 10.1097/00003086-200202000-00014 11937873

[44] M. Takao , T. Nishii , T. Sakai , H. Yoshikawa , and N. Sugano , “Iliosacral Screw Insertion Using CT‐3D‐Fluoroscopy Matching Navigation,” Injury 45, no. 6 (2014): 988–994. 24507831 10.1016/j.injury.2014.01.015

[45] F. R. Avilucea and M. L. Chip Routt, Jr. , “Blunt End Wire and Lateral Sacral View: A Technical Trick to Precisely Terminate Percutaneous Pelvic Brim Screw Fixation in the Posterior Ilium,” Journal of Orthopaedic Trauma 35, no. 1 (2021): e34–e36. 32467488 10.1097/BOT.0000000000001847

[46] J. G. Eastman and M. L. Chip Routt, Jr. , “Intramedullary Fixation Techniques for the Anterior Pelvic Ring,” Journal of Orthopaedic Trauma 32, no. S6 (2018): S4–s13. 10.1097/BOT.0000000000001250 30095675

